# Primary hyperoxaluria Type 1: A case report in an extended family with a novel AGXT gene mutation

**DOI:** 10.1097/MD.0000000000020371

**Published:** 2020-06-19

**Authors:** Mohamed W. Abukhatwah, Samia H. Almalki, Mohammed S. Althobaiti, Abdulla O. Alharbi, Najla K. Almalki, Naglaa M. Kamal

**Affiliations:** a Pediatric department, Alhada Armed Forces Hospital; b Nephrology department, Alhada Armed Forces Hospital, Taif, KSA; c Faculty of Medicine, Cairo University, Cairo, Egypt.

**Keywords:** AGXT gene, children, end stage renal disease, nephrolithiasis, novel mutation, primary hyperoxaluria, Saudi Arabia, urolithiasis

## Abstract

**Introduction:**

Primary hyperoxaluria type 1 (PH1) is a genetic autosomal recessively inherited disorder due to mutation in the alanine-glyoxylate aminotransferase (AGXT) gene. It usually presents in children with nephrolithiasis and/or nephrocalcinosis and progressive renal function impairment and end stage renal disease (ESRD).

**Patient concerns:**

A 13 years old Saudi boy with history of recurrent urolithiasis since the age of 2 years presented to us with picture of ESRD. He has strong family history of urolithiasis.

**Diagnosis:**

Working up the patient suggested the diagnosis of PH1 based on the typical clinical, laboratory, and imaging findings which was genetically proved by positive AXGT gene mutation. The mutation detected was not previously reported in literature. The mutation detected was not previously reported in literature. The novel mutation c. 799A>T p. (IIe267Phe) detected in our patient extend the spectrum of the known AGXT gene mutations.

**Interventions and Outcomes:**

Hemodialysis as a temporary step followed by renal transplantation which is the only cure.

**Conclusion:**

High index of suspicion of PH1 before ESRD should be considered in any patient who has recurrent urolithiasis since early life especially in presence of strong family history.

## 1 Introduction

Primary hyperoxalurias are a group of an inborn error of metabolism inherited as an autosomal recessive inheritance characterized by oxalate overproduction. There are 3 types of primary hyperoxalurias in which the underlying cause have been known. Primary hyperoxaluria type 1 (PH1), the most common form, has an estimated prevalence of 1 to 3 cases per 1 million population and an incidence rate of approximately 1 case per 120,000 live births per year in Europe.^[1,2]^

PH1 is caused by mutation in the alanine-glyoxylate aminotransferase (AGXT) gene and is characterized by endogenous oxalate overproduction secondary to AGXT enzyme deficiency with consequent impairment of renal functions and systemic oxalosis. More than 200 AGXT mutations have been described, spanning the entire gene. The 3 most common mutations, c. 508G>A (p.Gly170Arg), c. 33dupC (p.Lys12Glnfs∗156), and c. 731T>C (p.Ile244Thr), account for approximately 30%, 11%, and 6% of AGXT mutant alleles, respectively.^[3–5]^

We herein report a novel AGXT gene mutation in a Saudi male child diagnosed with PH1 with his extended family genetic study.

## 2 Case description

**Figure 1:**
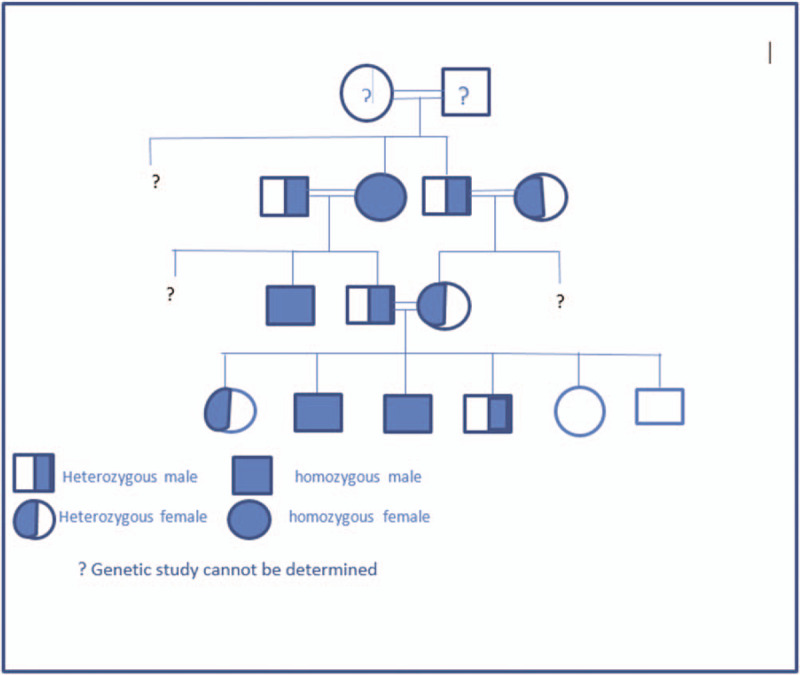
Family pedigree.

A 13 years old, Saudi boy, product of an uneventful pregnancy of consanguineous parents with positive family history of renal stones in his elder brother, his grandmother, and uncle from paternal side, presented to our care with picture of end stage renal disease (ESRD). Family pedigree is illustrated in Figure 1.

The patient has history of recurrent renal stones since early childhood when he presented to 1 hospital at the age of 2 years old with hypertension, severe metabolic acidosis, hyperkalemia, anuria, high serum creatinine (s.Cr) and high urinary calcium (Table 1). He was diagnosed as multiple bilateral renal stones necessitating nephrostomy, Extracorporeal Shock Wave Lithotripsy and surgical removal of a big stone from the Rt ureter. Analysis of the extracted stone revealed calcium oxalate.

**Table 1:**
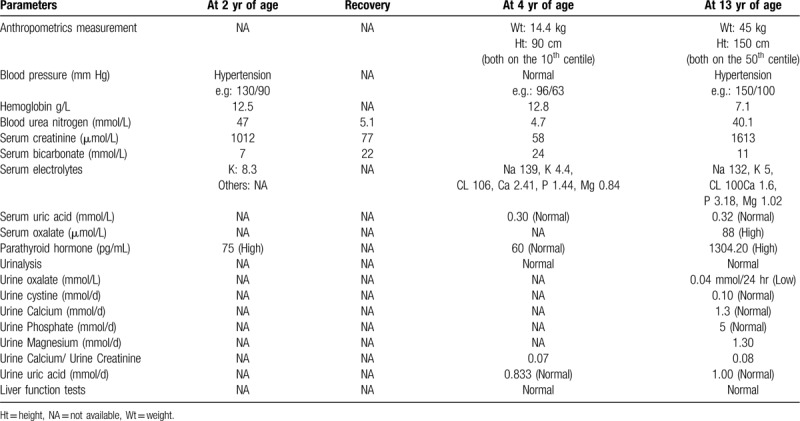
Patient's laboratory tests since initial presentation until diagnosis.

Parents missed follow-up of their child for 2 years. At age of 4 years, he was evaluated in another tertiary care hospital which revealed appropriate growth parameters, normal blood pressure and normal physical examination. All his laboratory tests were within normal limits except for elevated urinary oxalate 579 mmol/L (normal range: 44 to 344 mmol/L) as shown in Table 1. On follow-up at the age of 4 years, the patient was growing well with his weight and height on the 50^th^ and 25^th^, percentiles respectively. He had normal blood pressure and normal serum blood urea nitrogen, s.Cr and bicarbonate. His renal ultrasound (U/S) showed the right (Rt) kidney measuring 6.1x2.5 cm, the left (Lt) kidney measuring 8.4x3.4 cm with a 12 mm anteroposterior diameter of the renal pelvis. There was mild increase in echogenicity in both kidneys and no calculi were seen. Renal scan showed marked reduction of function and perfusion of the Rt kidney; the Lt kidney appeared to be normal. Split function of the Rt kidney was 14% and that of the Lt kidney was 88%. His nephrologist diagnosed him as resolved multiple calcium oxalate stones complicated by obstructive nephropathy and acute renal failure with residual impairment of Rt kidney functions. He started the patient on low oxalate diet and polycitra potassium for urine alkalization with advice for encouragement of excess oral fluids intake.

Although regular follow-up visits with pediatric urologist and nephrologist were recommended and highly emphasized but unfortunately the family missed follow-up again for around 7 years.

On 2016, at the age of 13 years old, he presented for the first time to our hospital with Lt flank pain, associated with nausea, vomiting, dysuria, and hematuria. He was hypertensive, thriving well but with Lt loin tenderness, facial puffiness and mild lower limb edema. There was no history suggestive of enteric hyperoxaluria with neither excessive intake of vitamin C nor oxalate rich diet or evidence of chronic malabsorption.

**Figure 2:**
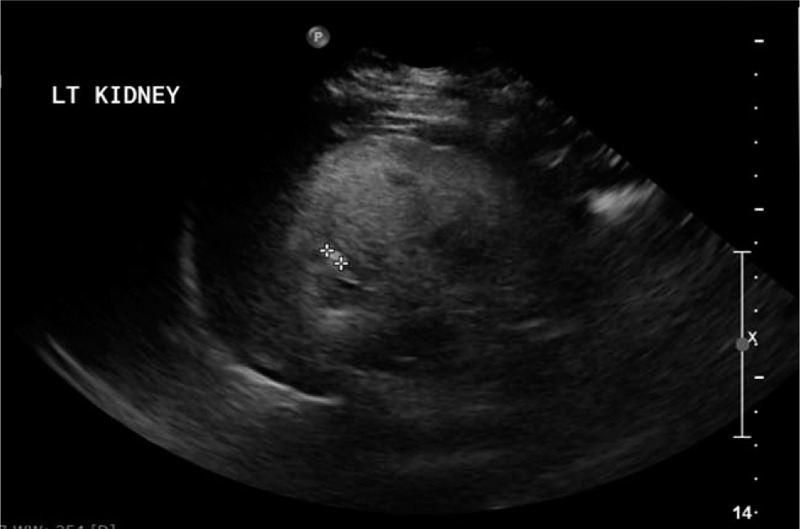
Ultrasound of left kidney obtained in sagittal view demonstrates stone measuring about 5 mm.

**Figure 3:**
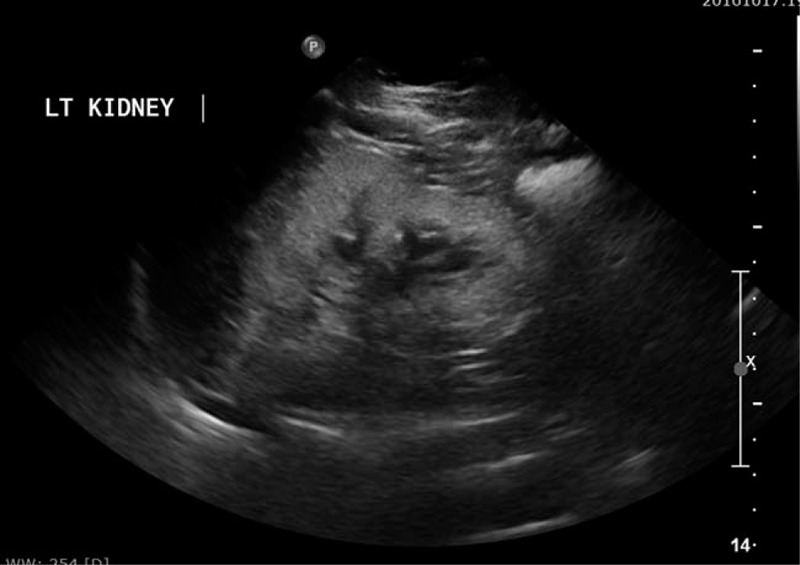
Ultrasound of left kidney obtained in sagittal view demonstrates increased echogenicity.

**Figure 4:**
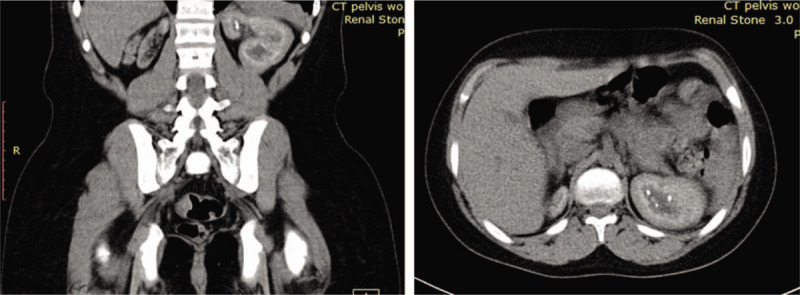
A & B: Coronal and sagittal noncontrast scan of the abdomen respectively showed small size right kidney, average size left kidney with multiple stones, and increased echogenicity.

His laboratory tests showed severe anemia, metabolic acidosis and markedly increased serum blood urea nitrogen and s.Cr with high phosphorus, magnesium, and parathyroid hormone levels (Table 1); while his kidney-ureter-bladder X-ray was non-conclusive. Urgent abdominal and pelvic ultrasound showed non-visualized Rt kidney and increased echogenicity of the Lt kidney with small stone measuring 5 mm (Fig. 2) and minimal hydronephrosis (Fig. 3). Urinary bladder showed average wall thickness with no stones. Non contrast computed tomography scan of the abdomen and pelvis showed small sized atrophic Rt kidney with multiple tiny stones 2 mm in diameter and increased density of the renal pyramids with associated fullness of the pelvicalyceal system (Fig. 4). The Lt kidney was average in size with multiple calyceal stone, the largest one at the upper calyx measuring 5 mm, with associated mild hydronephrosis and increased density of its pyramids (Fig. 4). Both ureters had normal course with no definite dense stones seen. The urinary bladder was improperly filled with no obvious dense stones seen. The diagnosis of ESRD with Lt renal stone was made and immediately planned for hemodialysis and Lt Double J stent insertion. PH1 was suggested as the underlying pathogenesis and it was worked-up. Its work-up revealed high serum oxalate, 88 μmol/L (normal: less than 5 μmol/L), and low urinary oxalate 0.04 mmol/24 h (normal level between 0.14 and 0.42). The diagnosis of PH1 was settled and molecular genetic testing of AGXT gene, after obtaining written informed consent, was requested to confirm diagnosis.

### 2.1 Molecular genetic analysis of the AGXT gene

**Figure 5:**
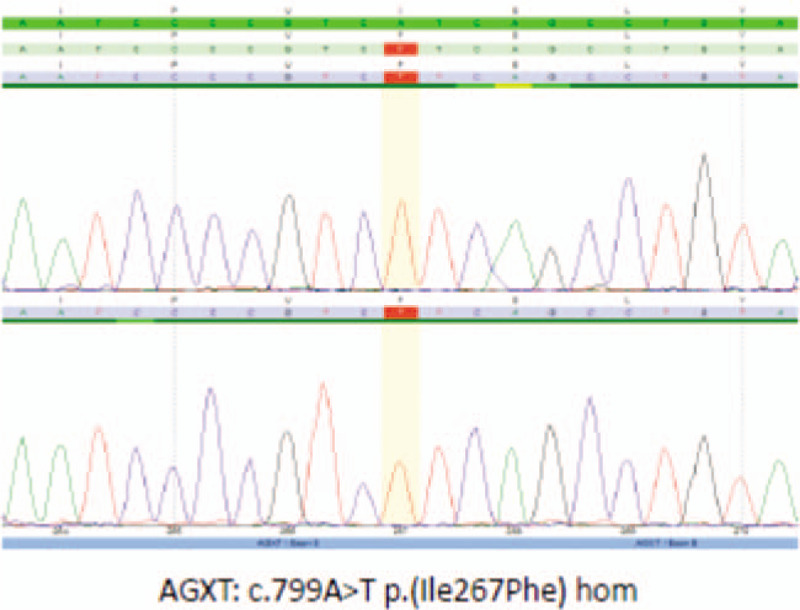
Molecular genetic analysis of the alanine-glyoxylate aminotransferase gene.

The 11 coding exons and the respective exon-intron boundaries of the AGXT gene on chromosome 2q37.3 (OMIM 604285) were amplified by polymerase chain reaction and sequenced directly. Resulting sequence data were compared with the reference sequence gene. Sequencing analysis revealed at position c.799 in exon 8 of the AGXT gene the nucleotide exchange A to T in homozygous state (c. 799A>T), resulting in a substitution of the evolutionarily conserved amino acid isoleucine to phenylalanine at position 267 of the protein sequence (p.(IIe267Phe)). To the best of our knowledge this mutation has neither been annotated in databases nor been described in the literature so far (Fig. 5).

His extended family was screened for the offending mutation described above and revealed that his parents and 2 siblings were carriers while 2 siblings were normal. His elder brother, uncle and grandpa to his father were diseased. Other family members from maternal and paternal sides were not feasible for genetic testing due to either refusal, living far, or being died (Fig. 1).

Currently, the patient is on hemodialysis 5 hours 5 times per week. He was evaluated for systemic oxalosis by multidisplinary team including cardiologist, ophthalmologist, and endocrinologist with normal electrocardiogram, echocardiography, ophthalmological examination, and thyroid functions. Joints X-rays were unremarkable. Health educator and social workers were involved in the care plan to ensure future family's compliance.

Liver-kidney transplantation is under processing in cooperation with higher centers with transplantation service.

## 3 Discussion

PH1 (OMIM#259900) is caused by a deficiency of the hepatic-specific peroxisomal enzyme AGXT, a pyridoxal 5′-phosphate–dependent enzyme that catalyzes the transformation of glyoxylate to glycine. This deficiency results in the buildup of glyoxylate and excessive production of both oxalate and glycolate.^[6]^

In Saudi Arabia, the incidence and prevalence of PH1 is not known, may be due to poor registration and lack of disease suspicion and follow-up. PH1 might be a rather not uncommon disease especially in our population because of high rates of consanguineous marriages.

Two reports of PH1 were released from the Kingdom of Saudi Arabia, 1 of 16 children released from King Faisal Specialist Hospital and Research Center^[7]^ and the other of 2 brothers released from King Abdul-Aziz Medical City.^[8]^

The age of presentation of PH1 is variable and depends on the disease severity, which may relate to the degree of hyperoxaluria and not the degree of enzyme activity. Fifteen percent of children become symptomatic before the age of 1 year and 50% before the age of 5 years. PH1 might present very early in life (infantile presentation) with nephrocalcinosis progressing rapidly to ESRD or might present in childhood with recurrent urolithiasis and progressive renal impairment or only occasional stone formation in adulthood or sometimes completely asymptomatic which was might be discovered only with family screening.^[9,10]^

Our patient started to have symptoms at 2 years and he was diagnosed at 13 years with 11 years delay interval between the onset of symptoms and diagnosis. We attribute that to lack of disease suspicion and rarity of the disease. Same was reported in Sanjad's study,^[7]^ with median age at presentation of 5 years (5 months–14 years) and 80% of their patients were diagnosed before the age of 10 years with 66% of them below the age of 5. The interval from the onset of symptoms to diagnosis ranged from 0 to 4 years, with a median of 18 months.^[7]^ In Majid's report, the 2 brothers started to have symptoms at age of 6 months and 2 years, respectively and they were diagnosed at that time.^[8]^

The late diagnosis of our patient after being already ESRD was also found in 4 patients of Sanjad's series, who presented with advanced chronic kidney injury and developed ESRD within 6 months.^[7]^

The reported index case presented with abdominal pain, nephrolithiasis and nephrocalcinosis which was consistent with the 7th, 12^th^, and 13^th^ patients of Sanjad's series.^[7]^

In this report, the patient had family members with symptomatic renal stones. While in Sanjad's study there were no symptomatic family members, they screened families of their cohort and 7 patients were identified.^[7]^

PH1 is a known cause of ESRD, it accounts for 1% to 2% of cases of pediatric ESRD, according to registries from Europe, the United States, and Japan,^[11]^ but it appears to be more prevalent in countries in which consanguineous marriages are common (with a prevalence of 10% or higher in some North African and Middle Eastern nations).^[12]^

The diagnosis depends on measurement of 24 hours of urine oxalate, serum oxalate level, enzymatic assay of AGXT catalytic activity by a liver biopsy, and genetic study of the AGXT gene, which can detect 50% to70% of mutations.^[13,14]^ High serum oxalate level and molecular genetic analysis proved the diagnosis in our patient with homozygous novel mutation in exon 8 of the AGXT gene on chromosome 2.q37.3, c.799A>T (p. IIe267Phe). To the best of our knowledge this mutation has neither been described before in the literature. Interestingly, the case described herein is the first report of molecular testing-proven childhood onset-PH1 from the Kingdom of Saudi Arabia with novel mutation.

Sanjad et al^[7]^ used quantitative urinary oxalate production or urine oxalate/creatinine ratio in their series while in 1 of the 2 brothers reported by Majid et al^[8]^ bone biopsy was used and genetic testing was used in the other 1.

Regarding PH1 management, maintaining high fluid intake, pyridoxine supplementation in those who are pyridoxine responsive, use of potassium or sodium citrate or neutral orthophosphate and magnesium oxide use to minimize stone formation are the main lines of treatment.^[13,14]^

Two patients in Sanjad's series^[7]^ and 1 of the 2 brothers in Majid's report^[8]^ were pyridoxine responsive and had complete response on pyridoxine therapy with normal renal function. The herein reported patient, did not receive pyridoxine in his past follow ups and he presented to us in ESRD where pyridoxine therapy response can not be assessed.

Organ transplantation in patients with PH1 is a controversial issue. Kidney transplantation has a considerably lower success rate than with other diseases.^[15]^

In Sanjad's series, a successful kidney transplant was achieved in a 15 years old patient while it failed with early rejection and death in another 2 younger patients.^[7]^ It seems that isolated renal transplantation should be reserved for older patients with less severe forms of PH1, and an insidious progression to chronic kidney failure.^[16,17]^

On the other hand; liver transplantation should be reserved for pyridoxine resistant patients with mild renal impairment and/or with sudden deterioration in renal function.^[18–20]^ On patient in Sanjad's series had isolated liver transplantation with normal kidney function at 4½ years follow up post-liver trans-plantation while 3 patients underwent combined liver-kidney transplantation with excellent outcome at 5 years follow up post-transplantation.^[7]^

We referred our patient for assessment for organ transplantation. This patient and his family highlight the need for physicians’ high index of suspicion for PH1 in any child who presents with nephrolithiasis and/or nephrocalcinosis or has a positive family history and/or parenteral consanguinity. Early diagnosis is crucial to allow intensive medical therapy which might delay disease progression to ESRD.

## 4 Conclusions

PH1 is a heterogeneous disease with variable expression in patients, even in family members with the same genotype. There has been speculation that environmental factors or modifier genes are responsible for the range of phenotypes within a single pedigree.

The case described herein for Saudi male child diagnosed with PH1 with novel mutation. This mutation may help to extend the spectrum of known AGXT mutations especially in our population since the incidence and prevalence of autosomal recessive diseases are expectedly high. Also, our report highlights the importance of early diagnosis and aggressive early treatment to prevent the disease progression and associated morbidity.

## Author contributions

MWA: diagnosed the patient, did investigations, followed up him and reviewed the manuscript.

NMK: reviewed literature and drafted the manuscript.

SA, AA, NA, and MA: collected data of the patient and drafted the manuscript.

All authors read and approved the final manuscript.

**Conceptualization:** Mohamed W Abukhatwa.

**Data curation:** Samia H Almalki, Mohammed S Althobaiti.

**Formal analysis:** Naglaa M Kamal.

**Investigation:** Mohamed W Abukhatwa.

**Methodology:** Mohamed W Abukhatwa.

**Project administration:** Mohamed W Abukhatwa.

**Supervision:** Mohamed W Abukhatwa, Naglaa M Kamal.

**Validation:** Mohamed W Abukhatwa.

**Writing – original draft:** Samia H Almalki, Mohammed S Althobaiti, Naglaa M Kamal.

**Writing – review & editing:** Mohamed W Abukhatwa, Naglaa M Kamal.

## References

[1] Cochat P Deloraine A Rotily M Epidemiology of primary hyperoxaluria type 1. Nephrol Dial Transplant 1995;10: Suppl. 8: 3–7. 10.1093/ndt/10.supp8.3 8592622

[2] van Woerden CS Groothoff JW Wanders RJ Primary hyperoxaluria type 1 in the Netherlands: prevalence and outcome. Nephrol Dial Transplant 2003;18:273–9. 12543880 10.1093/ndt/18.2.273

[3] Rumsby G Primary Hyperoxaluria Mutation Database; 2015. Available from: https://www.uclh.nhs.uk/OurServices/ServiceA-Z/PATH/PATHBIOMED/BIO/Pages/Phmdatabase.aspx. (Accessed on August 19, 2016).

[4] Rumsby G Williams E Coulter-Mackie M Evaluation of mutation screening as a first line test for the diagnosis of the primary hyperoxalurias. Kidney Int 2004;66:959–63. 15327387 10.1111/j.1523-1755.2004.00842.x

[5] Hopp K Cogal AG Bergstralh EJ Phenotype-genotype correlations and estimated carrier frequencies of primary hyperoxaluria. J Am Soc Nephrol 2015;26:2559–70. 25644115 10.1681/ASN.2014070698 PMC4587693

[6] Zhang X Roe SM Pearl LH Crystallization and preliminary crystallographic analysis of human alanine:glyoxylate aminotransferase and its polymorphic variants. Acta Crystallogr D Biol Crystallogr 2001;57:1936–7. 11717523 10.1107/s0907444901017334

[7] Sanjad SA Al-Abbad A Al-Sabban E Primary hyperoxaluria type I: an underestimated cause of nephrocalcinosis and chronic renal failure in Saudi Arabian children. Ann Saudi Med 1999;19:4–7. 17337975 10.5144/0256-4947.1999.4

[8] Alfadhel M Extreme intrafamilial variability of Saudi brothers with primary hyperoxaluria type 1. Ther Clin Risk Manag 2012;8:373. 22956877 10.2147/TCRM.S34954 PMC3431957

[9] Scheinman JI Primary hyperoxaluria: therapeutic strategies for the 90s. Kidney Int 1991;40:389–99. 1787639 10.1038/ki.1991.224

[10] Latta K Brodehl J Primary hyperoxaluria type I. Eur J Pediatr 1990;149:518–22. 2189732 10.1007/BF01957682

[11] Harambat J van Stralen KJ Espinosa L Characteristics and outcomes of children with primary oxalosis requiring renal replacement therapy. Clin J Am Soc Nephrol 2012;7:458–65. 22223608 10.2215/CJN.07430711 PMC3302673

[12] Danpure CJ Scriver CR Beaudet AL Sly WS Valle D Primary hyperoxaluria. McGraw-Hill, The Metabolic and Molecular Bases of Inherited Disease. New York: 2001.

[13] Kamoun A Lakhoua R End-stage renal disease of the Tunisian child: epidemiology, etiologies, and outcome. Pediatr Nephrol 1996;10:479–82. 8865247 10.1007/s004670050143

[14] Coulter-Mackie MB Rumsby G Genetic heterogeneity in primary hyperoxaluria type 1: impact on diagnosis. Mol Genet Metab 2004;83:38–46. 15464418 10.1016/j.ymgme.2004.08.009

[15] Broyer M Brunner FP Bryngen H Kidney transplantation in primary oxalosis: data from the EDTA Registry. Nephrol Dial Transplant 1990;5:332–6. 2115623 10.1093/ndt/5.5.332

[16] Thervet E Legendre CH Daudon M Is there a place for isolated renal transplantation in the treatment of hyperoxaluria type I? Experience from Paris. Nephrol Dial Transplant 1995;10:38–41. 10.1093/ndt/10.supp8.38 8592625

[17] Katz A Kim Y Scheinman J Long-term outcome of kidney transplantation in children with oxalosis. Transplant Proc 1989;21:2033–5. 2652665

[18] Latta K Jamieson NV Sheinman JI Selection of transplantation procedures and perioperative management in primary hyperoxaluria type I. Nephrol Dial Transplant 1995;10:53–7. 8592628 10.1093/ndt/10.supp8.53

[19] Coulthard MS Lodge JP Liver transplantation before advanced renal failure in primary hyperoxaluria type I. Pediatr Nephrol 1993;7:774. 8130105 10.1007/BF01213354

[20] Cochat P Schärer K Should liver transplantation be performed before advanced renal insufficiency in primary hyperoxaluria type I? Pediatr Nephrol 1993;7:212–8. 8476723 10.1007/BF00864408

